# Prognostic significance of reduced eGFR for kidney outcomes and all-cause mortality in older Japanese individuals with type 2 diabetes and normoalbuminuria: a single-center cohort study

**DOI:** 10.1007/s10157-026-02899-6

**Published:** 2026-06-08

**Authors:** Yui Yamamoto, Kaya Ishizawa, Tetsuya Babazono

**Affiliations:** Division of Diabetology and Metabolism, Department of Internal Medicine, Tokyo Women’s Medical University School of Medicine, 8-1 Kawada-Cho, Shinjuku-Ku, Tokyo, 162-8666 Japan

**Keywords:** eGFR, Chronic kidney disease, Elderly individuals, Normoalbuminuria, Type 2 diabetes

## Abstract

**Background:**

Because glomerular filtration rate (GFR) physiologically declines with age, a fixed estimated GFR (eGFR) threshold of < 60 mL/min/1.73 m^2^ to define chronic kidney disease may overestimate risk in older adults. We examined whether lower eGFR was associated with adverse outcomes in older Japanese adults with type 2 diabetes (T2D) and normoalbuminuria.

**Methods:**

We studied 901 Japanese adults aged ≥ 65 years with T2D and normoalbuminuria who underwent baseline assessment between 2012 and 2017. Participants were categorized by Japanese Society of Nephrology (JSN) eGFR as ≥ 90 (n = 41), 60–89 (n = 538), 45–59 (n = 251), and 15–44 mL/min/1.73 m^2^ (n = 71). Outcomes were a composite kidney outcome (≥ 30% eGFR decline, kidney replacement therapy, or kidney-related death), all-cause mortality, and eGFR slope. Analyses included Poisson regression, competing risk models, and restricted mean survival time. A sensitivity analysis recalculated eGFR using the 2021 CKD-EPI equation with a Japanese coefficient (CKD-EPI_J).

**Results:**

The mean age was 72.6 ± 5.5 years (SD) and 41.3% were women. Over median follow-up periods of 6.8 and 7.8 years, 245 kidney events and 48 deaths occurred, respectively. Baseline eGFR by the JSN equation was not independently associated with the composite kidney outcome, mortality, or eGFR slope. In contrast, the CKD-EPI_J equation reclassified 65.7% of participants with a JSN eGFR of 45–59 mL/min/1.73 m^2^ into a higher category, yielding a clearer gradient in the risk of kidney outcomes, whereas mortality risk estimates remained similar across categories.

**Conclusions:**

In this population, the association between lower eGFR and kidney outcomes differed by the equation used.

**Supplementary Information:**

The online version contains supplementary material available at https://doi.org/10.1007/s10157-026-02899-6.

## Introduction

Chronic kidney disease (CKD) commonly manifests as albuminuria, reduced kidney function, or both, regardless of diabetes status [1]. Recent studies have reported a temporal shift in CKD phenotypes among individuals with type 2 diabetes (T2D), characterized by a declining prevalence of albuminuria and an increasing prevalence of reduced estimated glomerular filtration rate (eGFR) [2–4]. Because aging leads to a physiological decline in GFR [5], this growing prevalence of reduced eGFR in T2D may partly reflect population aging, particularly in Japan, which has one of the most rapidly aging populations worldwide. Furthermore, the recent widespread use of renin–angiotensin system (RAS) blockers and sodium–glucose cotransporter 2 (SGLT2) inhibitors may have also modified CKD phenotypes; these agents reduce eGFR and albuminuria mainly by lowering intraglomerular pressure, which may contribute to long-term renoprotection [6, 7].

Both albuminuria and reduced GFR are independently associated with increased risk of end-stage kidney disease (ESKD) and mortality. Current guidelines define CKD using a fixed eGFR threshold of < 60 mL/min/1.73 m^2^ regardless of age [1]. However, this age-invariant threshold has been questioned because age-related GFR decline may lead to overdiagnosis and unnecessary disease labeling in older adults [5, 8]. It remains unclear whether reduced eGFR alone carries adverse prognostic implications in older adults with T2D, particularly those with normoalbuminuria. We therefore conducted a cohort study of older Japanese individuals with T2D and normoalbuminuria to examine the association between baseline eGFR and the risk of kidney outcomes, as well as all-cause mortality. To strengthen the interpretability of our findings, we applied multiple complementary statistical frameworks and also evaluated the potential impact of different eGFR estimating equations.

## Methods

### Study design and participants

**Fig. 1:**
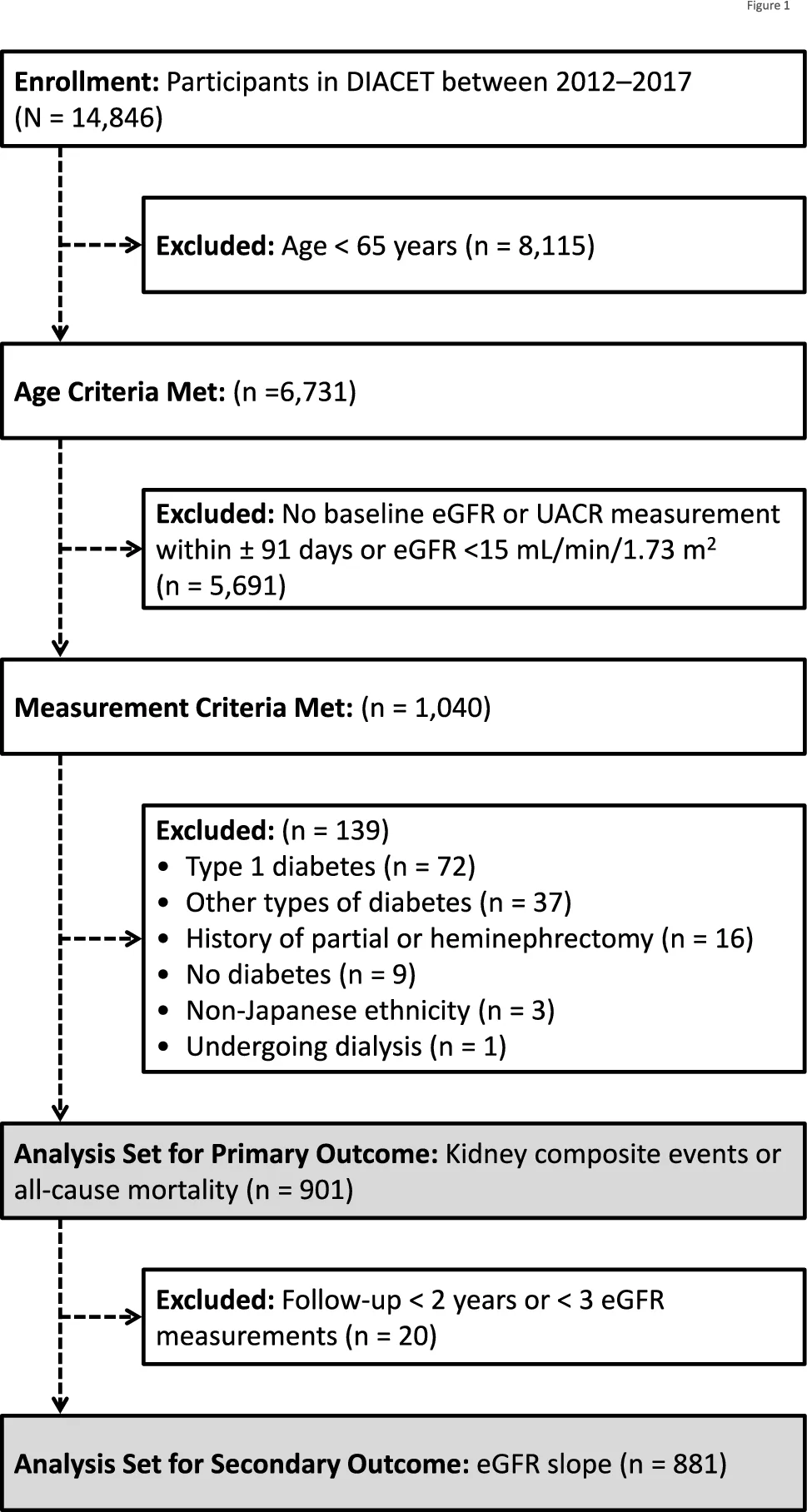
Flowchart of study participants selection. Of the 14,846 participants initially enrolled in the DIACET registry between 2012 and 2017, 901 met the eligibility criteria for the primary time-to-event analysis. For the secondary eGFR slope analysis, a subset of 881 participants with sufficient follow-up data (≥ 2 years and ≥ 3 eGFR measurements) was included. Abbreviations: DIACET, Diabetes Study from the Center of Tokyo Women’s Medical University; eGFR, Estimated glomerular filtration rate; UACR, Urinary albumin-to-creatinine ratio

This study analyzed data from the Diabetes Study from the Center of Tokyo Women’s Medical University (DIACET), a prospective cohort of individuals regularly attending the Diabetes Center, Tokyo Women’s Medical University [9]. We included participants who underwent the baseline assessment from 2012 to 2017 and met the following criteria: (1) Japanese ethnicity; (2) diagnosis of T2D; (3) age ≥ 65 years; (4) normoalbuminuria, defined as urinary albumin-to-creatinine ratio (UACR) < 30 mg/g, confirmed within ± 91 days of the baseline assessment; (5) eGFR measurement obtained within ± 91 days of both the baseline assessment and UACR measurement; and (6) baseline eGFR ≥ 15 mL/min/1.73 m^2^. We excluded individuals with a history of kidney replacement therapy (KRT; chronic dialysis or kidney transplantation) or partial/unilateral nephrectomy (Fig. 1). Participants were categorized by baseline eGFR (in mL/min/1.73 m^2^) according to the Kidney Disease: Improving Global Outcomes (KDIGO) guidelines [1]: G1 (≥ 90, reference), G2 (60–89), G3a (45–59), G3b (30–44), and G4 (15–29).

This study was conducted in accordance with the Declaration of Helsinki and is reported in accordance with the Strengthening the Reporting of Observational Studies in Epidemiology (STROBE) statement. The study protocol was approved by the Ethics Committee of Tokyo Women’s Medical University (Approval No. 2481-R), and written informed consent was obtained from all participants.

### Measurements and definitions

Baseline demographic and clinical characteristics were retrieved from structured questionnaires and electronic medical records. Depressive symptoms were assessed using the Patient Health Questionnaire-9 (PHQ-9) [9], with a score of ≥ 5 defined as the presence of depressive symptoms [10]. UACR was determined exclusively in first-morning urine samples to minimize intra-individual variation. Urinary albumin was measured by an immunoturbidimetric method; urinary and serum creatinine by an enzymatic assay. eGFR was calculated using the Japanese Society of Nephrology (JSN) creatinine-based equation [11].

### Follow-up and outcomes

The index date was defined as the date of the baseline assessment. The primary outcomes were a composite kidney outcome and all-cause mortality. The composite kidney outcome consisted of the first occurrence of: (1) a ≥ 30% decline in eGFR from baseline; (2) initiation of KRT; or (3) kidney-related death. Participants were followed from the index date until the earliest of the following: occurrence of an outcome event, death, lost to follow-up, or administrative censoring on November 30, 2025.

The secondary outcome was eGFR slope, estimated using all available serum creatinine measurements obtained during follow-up. For participants who initiated KRT or underwent partial/unilateral nephrectomy, subsequent eGFR data were censored at the time of these events to ensure the estimated slope reflected the trajectory of native kidney function.

### Statistical analysis

All statistical analyses were performed using SAS software version 9.4 (SAS Institute, Cary, NC, USA). Continuous variables were summarized as mean ± standard deviation (SD) or median (interquartile range [IQR]), and categorical variables were summarized as numbers (percentages). Model-based least-squares means (95% confidence intervals [CIs]) were estimated for continuous outcomes. Sample size was determined by the number of eligible participants available during the study period; no *a prior*i power calculation was performed.

Incidence rates (per 1000 person-years) were estimated using Poisson regression models [12]. For time-to-event outcomes, we accounted for competing risks by estimating the cumulative incidence function (CIF) and fitting Fine–Gray subdistribution hazard models [13]. Hazard-based inference was complemented by restricted mean survival time (RMST) analysis [14]. eGFR slope was estimated using linear mixed-effects models with random intercepts and slopes among participants with ≥ 2 years of follow-up and a minimum of 3 serum creatinine measurements [15].

Missing baseline data were addressed using multiple imputation, generating 100 imputed datasets [16]. The multivariable models were adjusted for the following covariates: sex, age (per 5-year increment), body mass index, diabetes duration (per 10-year increment), smoking status, history of cardiovascular disease, history of malignancy, presence of depressive symptoms (PHQ-9 score ≥ 5), log-transformed UACR, HbA1c, and hemoglobin.

Three sensitivity analyses were performed: 1) a complete-case analysis restricted to participants with complete baseline covariates; 2) variable selection using the least absolute shrinkage and selection operator (LASSO) within each imputed dataset [17]; and 3) a post hoc analysis in which all eGFR measurements, both at baseline for reclassification and throughout the follow-up period for outcome assessment, were recalculated using the 2021 CKD-EPI creatinine equation multiplied by the Japanese coefficient of 0.813 (CKD-EPI_J) [18]. The mean difference between baseline eGFR values calculated by the JSN and CKD-EPI_J equations was estimated with a 95% CI. On the basis of this CKD-EPI_J reclassification, we then re-estimated incidence rates, subdistribution hazard ratios, and RMST for the composite kidney outcome and all-cause mortality.

In accordance with the statement from the American Statistical Association, interpretation focused on effect estimates and 95% CIs rather than *P* values [19]. Detailed statistical procedures are provided in the Supplementary Statistical Methods.

## Results

### Baseline characteristics

Of the 14,846 DIACET participants assessed between 2012 and 2017, 901 met the eligibility criteria and were included in the primary analysis (Fig. 1). The cohort comprised 372 women and 529 men, with the mean age of 72.6 ± 5.5 years and the mean diabetes duration of 19.5 ± 10.5 years. The mean eGFR was 65.1 ± 14.4 mL/min/1.73 m^2^ and the median UACR was 7.4 (IQR, 4.9–12.8) mg/g. At baseline, 41, 538, 251, 68, and 3 participants were classified into eGFR categories G1, G2, G3a, G3b, and G4, respectively. Due to the small sample size in G4 (n = 3), G3b and G4 were combined into a single category (G3b–4, n = 71).

Participants with lower baseline eGFR were older, had a longer diabetes duration, exhibited a higher prevalence of comorbidities, and had lower hemoglobin levels. The proportion of women decreased with lower eGFR categories. UACR levels were comparable across the categories. Missing data for most baseline covariates were minimal (all < 10%), except for history of malignancy (24%) (Table 1).

**Table 1:** Clinical characteristics and laboratory findings across baseline eGFR category

eGFR category	G1 (n = 41)	G2 (n = 538)	G3a (n = 251)	G3b–4 (n = 71)	Total (N = 901)	Missing data, n (%)
Age, years	71.0 ± 4.1	71.7 ± 5.2	73.7 ± 5.5	76.0 ± 6.2	72.6 ± 5.5	0 (0.0)
Women, n (%)	19 (46.3)	229 (42.6)	93 (37.1)	31 (43.7)	372 (41.3)	0 (0.0)
Duration of diabetes, years	17.7 ± 10.4	19.3 ± 10.5	20.0 ± 10.7	20.1 ± 10.6	19.5 ± 10.5	23 (2.6)
BMI, kg/m^2^	22.4 ± 3.1	23.0 ± 3.3	23.7 ± 3.4	25.3 ± 3.8	23.4 ± 3.4	76 (8.4)
Diabetic neuropathy, n (%)	12 (32.4)	146 (29.7)	72 (29.9)	29 (43.9)	259 (31.0)	65 (7.2)
Diabetic retinopathy, n (%)	28 (75.7)	329 (66.9)	173 (71.8)	44 (66.7)	575 (68.7)	65 (7.2)
Cardiovascular disease, n (%)	7 (18.9)	58 (11.7)	40 (16.6)	20 (30.3)	125 (14.9)	63 (7.0)
Malignancy, n (%)	7 (22.6)	77 (18.9)	47 (24.1)	8 (15.7)	139 (20.3)	217 (24.1)
Depression, n (%)	11 (29.7)	124 (25.4)	49 (20.4)	17 (25.8)	201 (24.2)	69 (7.7)
Smoking use, n (%)	9 (24.3)	46 (9.4)	15 (6.3)	3 (4.6)	73 (8.8)	67 (7.4)
Alcohol use, n (%)	15 (40.5)	192 (39.1)	77 (32.1)	15 (22.7)	299 (35.9)	67 (7.4)
Oral antidiabetic agents, n (%)	29 (78.4)	363 (73.3)	191 (79.3)	54 (81.8)	637 (75.9)	62 (6.9)
Injectable medications, n (%)	9 (24.3)	132 (26.7)	76 (31.5)	27 (40.9)	244 (29.1)	62 (6.9)
Antihypertensive agents, n (%)	12 (32.4)	194 (39.6)	107 (44.4)	30 (45.5)	343 (41.1)	67 (7.4)
Lipid-lowering drugs, n (%)	11 (29.7)	195 (39.9)	109 (45.4)	33 (50.0)	348 (41.8)	69 (7.7)
HbA1c, %	7.3 ± 0.9	7.4 ± 0.9	7.3 ± 0.9	7.4 ± 1.0	7.3 ± 0.9	0 (0.0)
eGFR, mL/min/1.73 m^2^	98.9 ± 8.1	71.2 ± 7.7	53.9 ± 4.2	39.2 ± 4.7	65.1 ± 14.4	0 (0.0)
UACR, mg/g	8.3 (5.9 to 13.5)	7.4 (5.0 to 12.6)	7.2 (4.7 to 13.0)	7.5 (4.8 to 14.1)	7.4 (4.9 to 12.8)	0 (0.0)
Log (UACR [mg/g])	0.96 ± 0.25	0.90 ± 0.27	0.89 ± 0.28	0.92 ± 0.29	0.90 ± 0.26	0 (0.0)
Hemoglobin, g/dl	13.5 ± 1.4	13.7 ± 1.3	13.6 ± 1.3	13.1 ± 1.6	13.6 ± 1.3	49 (5.4)

Categorical variables are shown as n (%); continuous variables as mean ± SD, except for UACR, which is presented as median (interquartile range). The rightmost column shows the percentage of participants with missing data for each variable. Abbreviations: eGFR, Estimated glomerular filtration rate; n, Number of participants; BMI, Body mass index; HbA1c, Glycated hemoglobin; UACR, Urinary albumin-to-creatinine ratio

### Composite kidney outcome

**Fig. 2:**
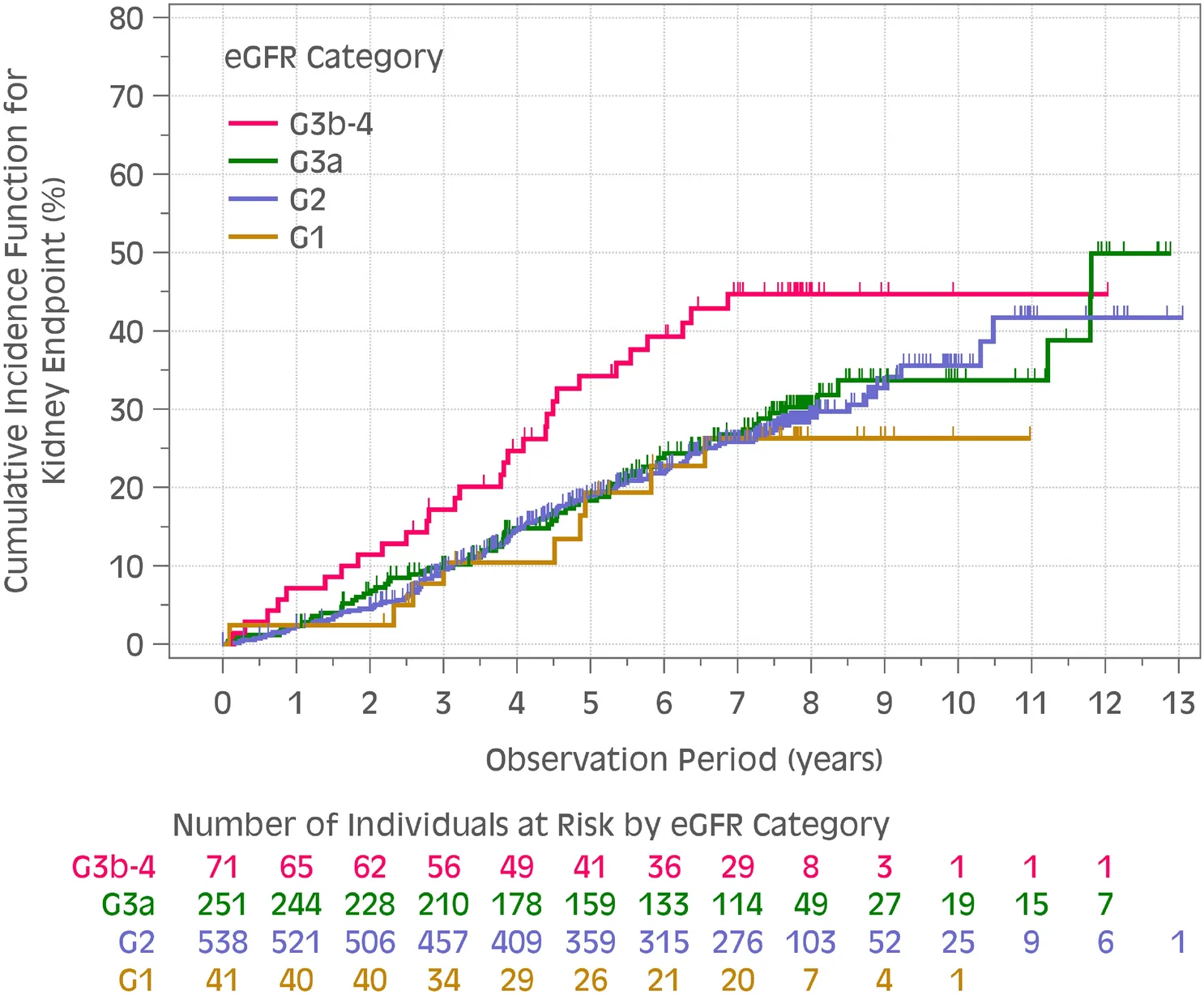
Cumulative incidence function of kidney endpoint across baseline eGFR category. Curves depict the cumulative incidence function estimated with the Fine–Gray method, treating non-kidney death and nephrectomy as competing events. The kidney endpoint was defined as the first occurrence of a ≥ 30% decline in eGFR from baseline, initiation of kidney replacement therapy, or kidney-related death. eGFR categories: G1 (≥ 90), G2 (60–89), G3a (45–59), and G3b–4 (15–44) mL/min/1.73 m^2^. Vertical tick marks indicate events. Numbers below the X-axis show individuals at risk at the start of each year; line colors correspond to the legend

Over a median follow-up of 6.8 years (IQR, 3.9 to 7.9), 245 composite kidney outcome events occurred. These events were driven exclusively by a ≥ 30% decline in eGFR, with no cases of KRT initiation, nephrectomy, or kidney-related deaths before the endpoint. Three non-kidney-related deaths occurred before the kidney event and were treated as competing events. The 10-year CIF for the composite kidney outcome was 26.3% (95% CI, 12.7 to 42.1) in G1, 35.6% (29.1 to 42.2) in G2, 33.7% (26.0 to 41.5) in G3a, and 44.7% (32.0 to 56.6) in G3b–4 (Fig. 2).

The multivariable-adjusted incidence rate was numerically higher in G3b–4 (67.01 per 1,000 person–years) than in G1 (57.39); however, the corresponding incidence rate ratio of 1.17 (95% CI, 0.57 to 2.41) did not exclude the null, with comparable findings for G2 and G3a (Table 2). In the multivariable Fine–Gray analysis, eGFR categories were not independently associated with the composite kidney endpoint; the subdistribution hazard ratios (SHRs) for G2 through G3b–4 versus G1 ranged from 1.27 to 1.33, with all 95% CIs crossing 1.00 (Table 2). The proportional subdistribution hazards assumption was satisfied for the eGFR category (Supplementary Table S1). Although diabetes duration, history of cardiovascular disease, and hemoglobin levels exhibited evidence of time-dependency, they were retained in the model to adjust for their time-averaged confounding effects. The 10-year RMST estimates were comparable across eGFR categories, with 95% CIs for the differences relative to G1 crossing zero (Table 2).

**Table 2:** Kidney outcomes and all-cause mortality across baseline eGFR category

eGFR category	n	IR (per 1000 person-years)		IRR versus G1		Fine-Gray competing risk Model		RMST (years)		Difference in RMST versus G1 (years)	
		IR	95% CI	IRR	95% CI	SHR	95% CI	Estimate	95% CI	Estimate	95% CI
Kidney endpoint											
G1	41	57.39	30.51 to 107.95	1.00	Ref	1.000	Ref	7.76	6.82 to 8.70	0.00	Ref
G2	538	59.72	44.14 to 80.80	1.04	0.56 to 1.94	1.274	0.640 to 2.534	7.48	6.95 to 8.01	− 0.27	− 1.16 to 0.61
G3a	251	55.66	38.75 to 79.94	0.97	0.50 to 1.87	1.202	0.582 to 2.481	7.63	7.03 to 8.23	− 0.13	− 1.07 to 0.82
G3b–4	71	67.01	42.23 to 106.34	1.17	0.57 to 2.41	1.326	0.604 to 2.996	7.10	6.19 to 8.02	− 0.66	− 1.86 to 0.55
All cause mortality											
G1	41	5.46	1.26 to 23.62	1.00	Ref	1.000	Ref	9.58	9.13 to 10.04	0.00	Ref
G2	538	5.28	2.60 to 10.75	0.97	0.22 to 4.24	0.677	0.155 to 2.953	9.67	9.44 to 9.89	0.08	− 0.33 to 0.50
G3a	251	8.50	3.97 to 18.18	1.56	0.34 to 7.04	0.760	0.163 to 3.547	9.56	9.30 to 9.83	− 0.02	− 0.48 to 0.43
G3b–4	71	14.34	5.96 to 34.50	2.63	0.54 to 12.67	0.987	0.204 to 4.776	9.49	9.08 to 9.91	− 0.09	− 0.64 to 0.47

Incidence rates (IR; per 1000 person-years) and incidence rate ratios (IRR) versus G1 were estimated using Poisson regression models. Subdistribution hazard ratios (SHRs) were obtained from Fine–Gray competing risk models. For both kidney endpoint and all-cause mortality, evidence of non-proportional subdistribution hazards was observed for some covariates (see Supplementary Tables S1 and S2). Restricted mean survival times (RMSTs) and the differences versus G1 were estimated at a truncation time (τ) of 10 years for both the composite kidney outcome and all-cause mortality. All analyses used multivariable models adjusted for the covariates described in the Methods section and were fitted after multiple imputation (*m* = 100). Abbreviations: eGFR, Estimated glomerular filtration rate; n, Number of participants; CI, Confidence interval; Ref, Reference category

### All-cause mortality

**Fig. 3:**
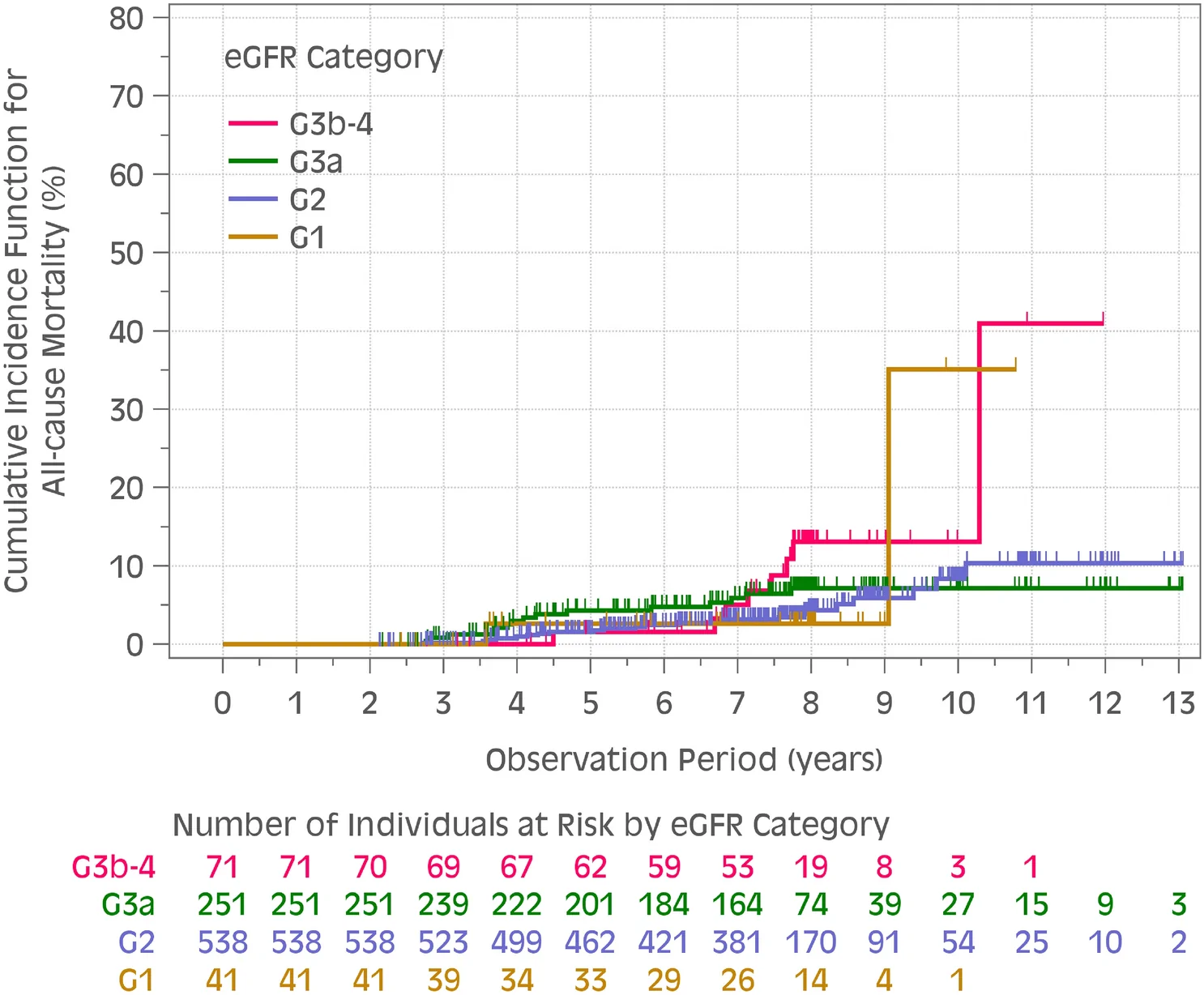
Cumulative incidence of all-cause mortality across baseline eGFR category. Curves show the cumulative incidence function estimated with the Fine–Gray method, treating initiation of kidney replacement therapy as a competing event. eGFR categories: G1 (≥ 90), G2 (60– 89), G3a (45–59), and G3b–4 (15–44) mL/min/1.73 m^2^. Vertical tick marks indicate deaths. Numbers below the X-axis denote individuals at risk at the start of each year; line colors correspond to the legend

During a median follow-up of 7.8 years (IQR, 6.3 to 8.1), 48 deaths occurred. Four participants initiated KRT before death; these cases were treated as competing events. The 10-year CIFs for mortality were 35.1% (95% CI, 0.3% to 83.1%) in G1, 8.4% (4.6% to 13.6%) in G2, 7.1% (4.1% to 11.2%) in G3a, and 13.1% (5.6% to 23.8%) in G3b–4 (Fig. 3). The CIF estimate for G1 was highly imprecise, as evidenced by the extremely wide 95% CI, reflecting the limited number of participants at risk toward the end of the follow-up period.

The multivariable-adjusted incidence rate ratio for G3b–4 compared with G1 was 2.63 (95% CI, 0.54 to 12.67), and the SHR was 0.99 (0.20 to 4.78) (Table 2). Consistent with the SHR, RMST differences were negligible. The 95% CIs across Poisson, Fine–Gray, and RMST analyses all included the null value. Regarding the Fine–Gray analysis for mortality, the proportional hazards assumption was upheld for the eGFR categories but was violated by certain covariates (diabetes duration, HbA1c, and depression) (Supplementary Table S2).

### eGFR slope

Among the 881 participants with a follow-up of ≥ 2 years and ≥ 3 serum creatinine measurements the median follow-up was 7.0 years (IQR, 5.4 to 7.4) (Fig. 1). In the linear mixed-effects models, the multivariable-adjusted estimated eGFR slope was comparable across baseline eGFR categories. Between-group differences were negligible, with all 95% CIs spanning zero (Table 3).

**Table 3:** eGFR slope across baseline eGFR category

eGFR category	n	eGFR slope (mL/min/1.73 m^2^/year)		Difference in eGFR slope from G1 (mL/min/1.73 m^2^/year)	
		Rate	95% CI	Rate	95% CI
Primary analyses (multiple imputation)					
G1	41	− 0.647	− 1.194 to −0.101	0.000	Ref
G2	527	− 0.860	− 1.044 to − 0.576	− 0.212	−0.752 to 0.327
G3a	243	− 0.788	− 1.113 to −0.462	− 0.140	− 0.708 to 0.427
G3b–4	70	− 0.822	−1.295 to − 0.349	−0.174	−0.845 to 0.496
Sensitivity analyses (complete case analyses)					
G1	29	− 0.616	− 1.293 to 0.061	0.000	Ref
G2	372	− 0.787	− 1.124 to −0.449	− 0.171	− 0.847 to 0.505
G3a	170	− 0.748	− 1.142 to −0.355	− 0.133	− 0.844 to 0.579
G3b–4	46	− 0.838	− 1.435 to −0.242	− 0.222	− 1.072 to 0.627
Sensitivity analyses (multiple imputation with LASSO-variable selection)					
G1	41	− 0.731	− 1.248 to − 0.214	0.000	Ref
G2	527	− 0.894	− 1.053 to − 0.736	− 0.164	− 0.697 to 0.370
G3a	243	− 0.843	− 1.069 to − 0.617	− 0.112	− 0.668 to 0.444
G3b–4	70	− 0.962	− 1.361 to − 0.562	− 0.231	− 0.878 to 0.417

Estimates were obtained from linear mixed-effects models with random intercepts and slopes. Primary analyses used multiple imputation (*m* = 100; missing-at-random) and adjusted for the prespecified covariate set described in the Methods section. Sensitivity analyses comprised: (i) complete-case analysis using the same covariates but restricted to fully observed records (no imputation); and (ii) models incorporating LASSO variable selection applied to the full set of covariates within each imputed dataset, which identified ‘depression’ as the sole covariate to be retained (selected in ≥ 50% of imputations). ‘Rate’ denotes the adjusted least-square mean eGFR slope (mL/min/1.73 m^2^/year; negative values indicate decline in eGFR). ‘Difference from G1’ refers to the adjusted difference compared with G1 Ref. Abbreviations: eGFR, Estimated glomerular filtration rate; n, Number of participants; CI = Confidence interval; LASSO, Least absolute shrinkage and selection operator; Ref, Reference category

### Sensitivity analyses

Complete-case analyses, as well as models utilizing LASSO-selected covariates, yielded results consistent with the primary multiple-imputation analyses across all outcomes, with widely overlapping 95% CIs (Tables 3, 4 and 5).

**Table 4:** Sensitivity analyses for composite kidney endpoint and all-cause mortality across baseline eGFR category using complete case analyses

eGFR category	n	IR (per 1000 person-years)		IRR versus G1		Fine-Gray competing risk model		RMST (years)		Difference in RMST versus G1 (years)	
		IR	95% CI	IRR	95% CI	SHR	95% CI	Estimate	95% CI	Estimate	95% CI
Composite kidney outcome											
G1	29	70.79	35.54 to 140.98	1.00	Ref	1.000	Ref	7.41	6.24 to 8.59	0.00	Ref
G2	381	56.32	39.63 to 80.03	0.80	0.40 to 1.59	1.033	0.451 to 2.364	7.53	6.94 to 8.11	0.11	− 1.01 to 1.24
G3a	174	54.47	35.65 to 83.23	0.77	0.37 to 1.60	1.035	0.435 to 2.465	7.59	6.93 to 8.24	0.17	− 1.02 to 1.37
G3b–4	47	66.90	39.45 to 113.45	0.95	0.42 to 2.13	1.142	0.440 to 2.967	7.07	6.01 to 8.14	− 0.34	− 1.83 to 1.16
All-cause mortality											
G1	29	6.55	1.47 to 29.20	1.00	Ref	1.000	Ref	9.46	8.83 to 10.08	0.00	Ref
G2	381	5.65	2.53 to 12.60	0.86	0.19 to3.88	0.603	0.126 to 2.877	9.61	9.34 to 9.88	0.16	− 0.42 to 0.74
G3a	174	7.55	3.12 to 18.26	1.15	0.24 to5.47	0.441	0.085 to 2.274	9.59	9.29 to 9.88	0.13	− 0.49 to 0.75
G3b–4	47	12.87	4.58 to 36.14	1.96	0.39 to 10.02	0.560	0.104 to 3.003	9.53	9.03 to 10.02	0.07	− 0.67 to 0.81

Analyses were restricted to participants with fully observed records for all covariates. Incidence rates (per 1,000 person-years) and incidence rate ratios versus G1 were estimated using Poisson regression models. Subdistribution hazard ratios (SHRs) were obtained from Fine–Gray competing risk models. For both kidney endpoint and all-cause mortality, evidence of non-proportional subdistribution hazards was observed for some covariates (see Supplementary Tables S1 and S2). Restricted mean survival times (RMSTs) and differences versus G1 were estimated at a truncation time (τ) of 10 years for both the composite kidney outcome and all-cause mortality. Abbreviations: CI, Confidence interval; eGFR, Estimated glomerular filtration rate; IR, Incidence rate; IRR, Incidence rate ratio; RMST, Restricted mean survival time; SHR, Subdistribution hazard ratio; UACR, Urine albumin-to-creatinine ratio

**Table 5:** Sensitivity analyses for composite kidney endpoint and all-cause mortality across baseline eGFR category using multiple imputation with LASSO-variable selection

eGFR category	n	IR (per 1000 person-years)		IRR versus G1		Fine-Gray competing risk model		RMST (years)		Difference in RMST versus G1 (years)	
		IR	95% CI	IRR	95% CI	SHR	95% CI	Estimate	95% CI	Estimate	95% CI
Composite kidney outcome											
G1	41	44.25	24.30 to 80.59	1.00	Ref	1.000	Ref	8.19	7.29 to 9.08	0.00	Ref
G2	538	45.12	37.79 to 53.86	1.02	0.55 to 1.89	1.245	0.631 to 2.453	7.96	7.67 to 8.25	− 0.23	− 1.15 to 0.70
G3a	251	46.17	35.74 to 59.65	1.04	0.55 to 1.99	1.293	0.638 to 2.619	7.98	7.58 to 8.38	− 0.21	− 1.18 to 0.77
G3b–4	71	65.03	44.50 to 95.01	1.47	0.73 to 2.96	1.694	0.785 to 3.654	7.19	6.38 to 8.00	− 1.00	− 2.21 to 0.22
All-cause mortality											
G1	41	6.92	1.71 to 27.90	1.00	Ref	0.000	Ref	9.63	9.21 to 10.04	0.00	Ref
G2	538	5.64	3.72 to 8.56	0.82	0.19 to 3.48	0.616	0.145 to 2.622	9.75	9.65 to 9.85	0.12	− 0.30 to 0.54
G3a	251	9.43	5.92 to 15.02	1.36	0.31 to 5.93	0.728	0.160 to 3.309	9.63	9.45 to 9.81	− 0.00	− 0.46 to 0.46
G3b–4	71	16.65	8.90 to 31.14	2.41	0.52 to 11.09	1.028	0.213 to 4.971	9.52	9.14 to 9.90	− 0.11	− 0.69 to 0.47

Estimates were derived from multivariable models applied to multiple imputation datasets (*m* = 100). The least absolute shrinkage and selection operator (LASSO) method was used to identify prognostic covariates. Covariates selected in ≥ 50% of imputations and adjusted for were age, diabetes duration, depression, hemoglobin, and log-transformed UACR for the kidney endpoint, and age, diabetes duration, and hemoglobin for all-cause mortality. Incidence rates (per 1,000 person-years) and incidence rate ratios versus G1 were estimated from Poisson models. Subdistribution hazard ratios (SHRs) were obtained from Fine–Gray competing risk models. For both kidney endpoint and all-cause mortality, evidence of non-proportional subdistribution hazards was observed for some covariates (see Supplementary Tables S1 and S2). Restricted mean survival times (RMSTs) and differences versus G1 were estimated at a truncation time (τ) of 10 years. Abbreviations: CI, Confidence interval; eGFR, Estimated glomerular filtration rate; IRR, Incidence rate ratio; RMST, Restricted mean survival time; SHR, Subdistribution hazard ratio; UACR, Urine albumin-to-creatinine ratio

In the third sensitivity analysis using the CKD-EPI_J equation, the mean baseline eGFR was higher (70.0 ± 11.0 mL/min/1.73 m^2^) than that calculated using the JSN Eq. (65.1 ± 14.4 mL/min/1.73 m^2^), with a mean difference of 4.9 mL/min/1.73 m^2^ (95% CI, 4.5 to 5.4) (Supplementary Figure S1). Following this upward shift, no participants remained in G1; rather, 744, 124, and 33 participants were stratified into G2, G3a, and G3b–4, respectively (Supplementary Fig. 1, Supplementary Table S3). Their baseline characteristics are summarized in Supplementary Table S4. The cumulative incidence curves for the composite kidney outcome demonstrated a clear and early separation of G3b–4 from G2 and G3a (Supplementary Figure S2). Using G2 as the reference, the multivariable-adjusted risk for the composite kidney outcome was higher in G3b–4, as the 95% CIs for the IRR, SHR, and RMST difference did not include the null values (Supplementary Table S5). Conversely, for all-cause mortality, the cumulative incidence curves showed no distinct separation among the groups (Supplementary Figure S3), with the 95% CIs for both the SHR and RMST difference including the null values (Supplementary Table S5).

## Discussion

In this cohort of older Japanese individuals with T2D and normoalbuminuria, lower baseline eGFR calculated using the JSN equation was not independently associated with an increased risk of the composite kidney outcome, all-cause mortality, or a steeper eGFR slope. These findings were consistent across multiple statistical approaches. However, a sensitivity analysis using the Japanese coefficient-modified CKD-EPI equation for eGFR (CKD-EPI_J) showed a different risk profile. After reclassification with the CKD-EPI_J equation, the risk for the composite kidney outcome was substantially higher in the advanced G3b–4 category. In contrast, the association with all-cause mortality remained weak, with estimates largely including the null, consistent with the results obtained using the JSN equation. Taken together, these results indicate that in older adults with normoalbuminuria, the association between lower eGFR for kidney outcome differed according to the estimating equation used.

The discrepancy in risk prediction suggests a reclassification phenomenon arising by the mathematical formulations of the equations. The JSN equation assigns substantial weight to age [11], which may classify age-related physiological decline as impaired kidney function in older populations. Historically, the transition from the Modification of Diet in Renal Disease (MDRD) equation to the CKD-EPI equation improved risk prediction by reducing false-positive CKD diagnoses [20]. Similarly, our results suggest that the JSN equation, modified from the MDRD equation for the Japanese population, may classify a substantial number of relatively low-risk older individuals into lower eGFR categories, thereby attenuating the observed prognostic risk toward the null. By contrast, the CKD-EPI_J equation appeared to identify a smaller subgroup at higher risk of kidney events.

Our findings should be contextualized within previous Japanese cohort studies of individuals with T2D [21, 22]. The multicenter study by Wada et al. (N = 4328) showed that while reduced eGFR combined with albuminuria strongly predicted kidney events, reduced eGFR with normoalbuminuria lacked a clear association [21]. This lack of a clear association in the normoalbuminuric subgroup is broadly consistent with our current JSN-based primary analysis. By contrast, our earlier single-center study (N = 8320) found that normoalbuminuric kidney insufficiency was associated with higher kidney and mortality risks [22]. However, compared with that study, the present analysis focused exclusively on older adults and used a less stringent kidney endpoint (≥ 30% decline in the present study versus ≥ 50% in the earlier study).

Several factors likely explain these differing risk associations. First, in our older normoalbuminuric population, modest eGFR reductions classified by the JSN equation may largely reflect age-related physiological decline, thereby attenuating the observed risk gradient. The clearer gradient unmasked by the CKD-EPI_J equation supports this. Second, the recruitment periods for the earlier studies (1985–2010 and 2003–2017, respectively) largely preceded our current baseline (2012–2017). Major advances in diabetes and CKD management since those periods may have favorably altered the clinical trajectory of the normoalbuminuric CKD phenotype in our present cohort, further contributing to the attenuated risk associations.

These results of the present study have important implications for current CKD definitions and clinical practice. Although international guidelines currently apply a fixed eGFR threshold of < 60 mL/min/1.73 m^2^ to define CKD regardless of age [1], there is an ongoing debate regarding whether mild-to-moderate GFR reductions in older adults represent true pathology or merely physiological senescence [5, 8]. Overdiagnosis in this population can lead to unnecessary interventions, psychological burden, and increased healthcare expenditures. Our results suggest that heavily age-weighted equations, such as the JSN equation, reduce prognostic discrimination in older adults, whereas the CKD-EPI_J equation better preserves kidney risk gradients. Importantly, the clearer association observed with CKD-EPI_J suggests that equation choice may affect the clinical interpretation of reduced eGFR in older adults. Although our findings imply that reduced eGFR in this population may partly reflect physiological aging rather than progressive kidney disease, this interpretation should be drawn cautiously. Given the single-center nature of our cohort, these findings may not be directly generalizable to broader populations, and further studies are needed to definitively support age-adapted risk assessments. Therefore, reduced eGFR in older adults without albuminuria should be interpreted carefully, and clinical assessment should not rely solely on a fixed eGFR threshold.

Our study has several limitations. First, because this was a single-center study of older Japanese individuals, generalizability of the findings to other clinical settings and populations should be confirmed. Second, the number of outcomes was modest, and some eGFR categories, particularly the reference group (eGFR ≥ 90 mL/min/1.73 m^2^), included few participants. In addition, all kidney outcomes were based on eGFR decline, with no hard kidney endpoints such as kidney replacement therapy or kidney-related death. These features may have reduced statistical precision and limited the direct extrapolation of our findings to hard kidney outcomes. Third, measured GFR was not available; therefore, we could not determine which creatinine-based equation more accurately reflected actual kidney function. Fourth, albuminuria was assessed only once at baseline. Given the intra-individual variability of albumin excretion, this may have introduced misclassification bias regarding the true normoalbuminuric phenotype. However, the use of first-morning urine samples may have reduced variability in albuminuria measurement. Fifth, the requirement of at least 2 years of follow-up and three or more serum creatinine measurements for the eGFR slope analysis may have introduced survivor bias. These criteria were prespecified to reduce the influence of short-term eGFR fluctuations. Finally, we lacked data on pharmacotherapy data at baseline and during follow-up; therefore, we could not account for potential confounding by treatment, including time-varying changes in therapy.

Despite these limitations, our study has several notable strengths. First, it focused on an underrepresented yet clinically relevant population: older adults with type 2 diabetes and normoalbuminuria. Second, we used multiple complementary statistical approaches, together with several sensitivity analyses, to assess the robustness of the findings. Third, the relatively long follow-up period allowed more reliable estimation of eGFR slopes.

## Conclusion

Our study showed that among older Japanese adults with T2D and normoalbuminuria, the association between lower baseline eGFR and kidney outcomes differed according to the estimating equation used, whereas no clear association was observed for all-cause mortality. These findings suggest that equation choice should be considered when interpreting reduced eGFR in this population, especially in the absence of albuminuria.

## Supplementary Information

Below is the link to the electronic supplementary material.

Fig. S1 Correlation between baseline eGFR values calculated by the JSN and CKD-EPI_J equations. Scatter plot comparing baseline estimated glomerular filtration rate (eGFR) values (N = 901) calculated using the Japanese Society of Nephrology (JSN) equation and the Japanese-coefficient-modified Chronic Kidney Disease Epidemiology Collaboration (CKD-EPI_J) equation. Fig. S2 Cumulative incidence function of kidney endpoint across baseline eGFR categories using the CKD-EPI_J equation. Curves depict the cumulative incidence function estimated with the Fine–Gray method, treating non-kidney death and nephrectomy as competing events. The kidney endpoint was defined as the first occurrence of a ≥30% decline in eGFR from baseline, initiation of kidney replacement therapy, or kidney-related death. Baseline eGFR was calculated using the CKD-EPI_J equation. eGFR categories: G2 (60–89), G3a (45–59), and G3b–4 (15–44) mL/min/1.73 m²; no participants were classified as G1 (≥90 mL/min/1.73 m²). Vertical tick marks indicate events. Numbers below the X-axis show individuals at risk at the start of each observation year; line colors correspond to the legend. Fig. S3 Cumulative incidence function of all-cause mortality across baseline eGFR categories using the CKD-EPI_J equation. Curves show the cumulative incidence function estimated with the Fine–Gray method, treating initiation of kidney replacement therapy as a competing event. Baseline eGFR was calculated using the CKD-EPI_J equation. eGFR categories: G2 (60–89), G3a (45–59), and G3b–4 (15–44) mL/min/1.73 m². Vertical tick marks indicate deaths. Numbers below the X-axis denote individuals at risk at the start of each observation year; line colors correspond to the legend. Supplementary file1 (PPTX 836 KB)

Supplementary file2 (DOCX 26 KB)

Supplementary file3 (PPTX 73 KB)

## Data Availability

The data used in this study are not available publicly.
