# Supplementary material for: Prognostic significance of reduced eGFR for kidney outcomes and all-cause mortality in older Japanese individuals with type 2 diabetes and normoalbuminuria: a single-center cohort study
Source: Clin Exp Nephrol. 2026 Jun 8;30(10):1441–50. doi: 10.1007/s10157-026-02899-6 (PMC13582078; doi:10.1007/s10157-026-02899-6)
Supplement: Supplementary file 1 [file 10157_2026_2899_MOESM2_ESM.docx]

**Supplementary Statistical Methods**

1. **Poisson regression for incidence rates**

Incidence rates (per 1,000 person-year) and incidence rate ratios were estimated using multivariable Poisson regression with log(person-time) as an offset, with the baseline eGFR G1 category serving as the reference group [1]. Person-time accrued from the index date until the occurrence of the event of interest, death, or the end of follow-up. Standard errors were adjusted for overdispersion using the Pearson scale parameter to ensure robust inference in the presence of greater-than-expected variance.

2. **Competing‐risks models for time-to-event outcomes**

Generally, in longitudinal studies of older adults, treating deaths or other terminal events as non-informative censoring can lead to a significant overestimation of the cumulative incidence of the primary outcome. To address this, we estimated the cumulative incidence function (CIF) and employed multivariable Fine–Gray subdistribution hazard models. Such approaches are recommended in nephrology settings where competing risks are common [2].

- **For the composite kidney outcomes:** Non-kidney death and partial/unilateral nephrectomy were treated as competing events, as these preclude the development of the predefined kidney endpoint.
- **For all-cause mortality:** Initiation of kidney replacement therapy (KRT) was treated as a competing event to focus on mortality occurring prior to end-stage kidney disease.

3. **Checking proportional subdistribution hazards**

The proportional subdistribution hazards assumption was rigorously verified by adding time-dependent covariates (interaction terms between each covariate and the log of time) into the model. A 95% CI for the interaction coefficient that did not include zero was interpreted as evidence of time dependence (i.e., non-proportionality) [3]. As a more formal, complementary approach for the Fine–Gray model, diagnostics based on cumulative sums of residuals have been proposed to probe violations of proportionality [4].

When evidence of non-proportionality was observed for potential confounders, these covariates were retained in the multivariable Fine–Gray models without introducing time-by-covariate interaction terms. In this setting, the estimated subdistribution hazard ratios were interpreted as summary measures reflecting time-averaged effects over the follow-up period. This interpretation draws on the general property of proportional hazards models where coefficients represent weighted averages of time-varying effects [5], a concept that has been further formalized for subdistribution hazards in competing risks settings [6].

4. **Restricted mean survival time (RMST) as a complementary estimand**

RMST is an increasingly used measure in contemporary survival analysis [7,8]. Although hazard ratios have been widely used, they can be hard to interpret and may be sensitive to violations of proportional hazards. We therefore complemented hazard-based inference with RMST, which provides a clinically intuitive measure of the average event-free time up to a specific time point (τ). RMST provides an absolute, model-robust measure that remains valid when proportional hazards do not hold. The truncation time point (τ) was restricted to be within the observable follow-up range of groups, specifically not exceeding the minimum of the largest observed follow-up times, to ensure the stability of the Kaplan–Meier estimates.

5. **Linear mixed-effects models for eGFR slope**

The annual rate of change in eGFR (slope) was estimated using linear mixed-effects models with random intercepts and slopes to account for both between-individual differences and within-individual correlation of repeated measurements, using all available longitudinal eGFR measurements derived from serum creatinine. To differentiate true chronic kidney function decline from acute fluctuations or transient noise, we restricted this analysis to participants with at least 2 years of follow-up and a minimum of 3 serum creatinine measurements [9].

6. **Multiple imputation for missing data**

Given missingness in several baseline covariates (**Table 1**), we used multiple imputation under a missing-at-random (MAR) assumption to reduce potential bias and loss of precision, generating 100 imputed datasets using a fully conditional specification [10]. The imputation model incorporated all variables used in the analyses, including the outcome variable, to preserve associations between covariates and the outcomes and support validity under MAR, as recommended in the methodological literature [11]. Each imputed dataset was analyzed using the prespecified multivariable models, and parameter estimates were combined using Rubin’s rules to reflect within- and between-imputation uncertainty. Because multiplicity-adjusted procedures such as Dunnett’s test are not straightforward to apply after pooling results across multiple imputations, we made no formal adjustment for multiple comparisons; interpretation emphasized point estimates and 95% CIs rather than hypothesis testing.

Complete-case analysis is unbiased only under the more restrictive missing completely at random (MCAR) assumption and otherwise risks loss of efficiency and biased estimates. Therefore, multiple imputation served as the primary analytic approach, with complete-case models used as a sensitivity analysis to evaluate robustness.

7. **Variable selection in the presence of imputation**

To assess robustness and mitigate potential overfitting, we applied the least absolute shrinkage and selection operator (LASSO) separately within each imputed dataset [12]. LASSO is a penalized regression method that performs shrinkage and variable selection simultaneously. We then retained covariates selected in ≥50% of the imputed datasets and refitted the multivariable models using this parsimonious set. This majority-vote approach for LASSO across multiply imputed data is a validated workflow for achieving parsimonious yet robust inference [13].

8. **Statistical Inference and Reporting Policy**

Following the American Statistical Association (ASA) statement on moving to a world beyond "p < 0.05" [14], we deliberately refrained from using P-values for any statistical inference in this study. Instead, our reporting and interpretation focus exclusively on the magnitude of effect sizes (e.g., hazard ratios, incidence rate ratios, and RMST differences) and the precision of these estimates, as reflected by their 95% confidence intervals (CIs). This approach ensures that clinical and biological importance is prioritized over arbitrary thresholds of statistical significance. The 95% CIs are intended to show the range of effect sizes that are reasonably compatible with our data, and all findings are discussed in terms of their clinical relevance and the strength of the observed associations.

**References for Supplementary Statistical Methods**

1. Frome EL. The analysis of rates using Poisson regression models. Biometrics. 1983;39:665–74.
2. Noordzij M, Leffondré K, van Stralen KJ, Zoccali C, Dekker FW, Jager KJ. When do we need competing risks methods in nephrology? Nephrol Dial Transplant. 2013;28:2670–7.
3. Bellera CA, MacGrogan G, Debled M, de Lara C, Brouste V, Mathoulin-Pélissier S. Variables with time-varying effects and the Cox model: some statistical concepts illustrated with a prognostic factor study in breast cancer. BMC Med Res Methodol. 2010;10:20.
4. Li J, Scheike TH, Zhang MJ. Checking Fine and Gray models with cumulative sums of residuals. Lifetime Data Anal. 2015;21:197–217.
5. Schemper M. Cox analysis of survival data with non-proportional hazard functions. Statistician. 1992;41:455–65.
6. Diao G, Ibrahim JG. Quantifying time-varying cause-specific hazard and subdistribution hazard ratios with competing risks data. Clin Trials. 2019;16:363–74.
7. Royston P, Parmar MK. Restricted mean survival time: an alternative to the hazard ratio for the design and analysis of randomized trials with a time-to-event outcome. BMC Med Res Methodol.2013;13:152
8. Peng ZY, Yang CT, Kuo S, Wu CH, Lin WH, Ou HT. RMST analysis for cardiorenal outcomes with SGLT2i. JAMA Netw Open. 2022;5:e2246928.
9. Itano S, Kanda E, Nagasu H, Nangaku M, Kashihara N. eGFR slope reliability in Japanese CKD. Clin Exp Nephrol. 2023;27:847–56.
10. Sterne JAC, White IR, Carlin JB, Spratt M, Royston P, Kenward MG, Wood AM, Carpenter JR. Multiple imputation in epidemiological research. BMJ. 2009;338:b2393.
11. Austin PC, White IR, Lee DS, van Buuren S. Missing data in clinical research: A tutorial on multiple imputation. Can J Cardiol 2021;37:1322–31
12. Tibshirani R. Regression shrinkage and selection via the LASSO. J R Stat Soc B. 1996;58:267–88.
13. Musoro JZ, Zwinderman AH, Puhan MA, Ter Riet G. Validation of prediction models based on LASSO with multiply imputed data. BMC Med Res Methodol. 2014;14:116.
14. Wasserstein RL, Lazar NA. The ASA’s statement on P-values. Am Stat. 2016;70:129–33
